# Targeting yes-associated protein to overcome imatinib resistance in gastrointestinal stromal tumor drug-tolerant persister cells

**DOI:** 10.1007/s10120-025-01657-z

**Published:** 2025-09-08

**Authors:** Takashi Yokouchi, Tsuyoshi Takahashi, Toshirou Nishida, Koji Tanaka, Yukinori Kurokawa, Kazuyoshi Yamamoto, Takuro Saito, Takaomi Hagi, Kota Momose, Kotaro Yamashita, Tomoki Makino, Kunihiko Kawai, Satoshi Serada, Minoru Fujimoto, Seiichi Hirota, Kiyokazu Nakajima, Tetsuji Naka, Hidetoshi Eguchi, Yuichiro Doki

**Affiliations:** 1 Department of Gastroenterological Surgery, The University of Osaka Graduate School of Medicine, 2-2, Yamadaoka, Suita, Osaka, 565-0871 Japan; 2 Department of Surgery, Japan Community Healthcare Organization Osaka Hospital, Osaka, Japan; 3 Institute for Biomedical Sciences Molecular Pathophysiology, Iwate Medical University School of Medicine, Yahaba, Japan; 4 Division of Allergy and Rheumatology, Department of Internal Medicine, Iwate Medical University School of Medicine, Yahaba, Japan; 5 Department of Surgical Pathology, Hyogo Medical University School of Medicine, Nishinomiya, Japan

**Keywords:** GIST, Drug-tolerant persister cells, Yes-associated protein

## Abstract

**Background:**

The tyrosine kinase inhibitor (TKI) imatinib targets KIT and PDGFRA, offering significant therapeutic benefits in advanced gastrointestinal stromal tumors (GISTs). However, the high rate of recurrence following treatment discontinuation suggests that drug-tolerant persister cells (DTPs) may contribute to therapy resistance. Elucidating the mechanisms underlying DTP survival is critical for the development of curative strategies. This study aimed to investigate the role of yes-associated protein (YAP) in DTP survival and to evaluate the efficacy of combining imatinib with YAP inhibitors as a potential therapeutic approach.

**Methods:**

Imatinib-sensitive GIST cell lines were treated with imatinib to generate DTPs. YAP activity was assessed via western blotting, fluorescence immunostaining, and nuclear-cytoplasmic fractionation. Proliferation and apoptosis assays were conducted to evaluate sensitivity to YAP inhibitors, such as verteporfin. Xenograft mouse models were used to assess the efficacy of combination therapy with imatinib and verteporfin.

**Results:**

DTPs exhibited increased nuclear localization and activity of YAP, which was reversible upon imatinib withdrawal. YAP inhibitors reduced nuclear YAP levels and showed greater efficacy in DTPs than in parental cells. Combination therapy with imatinib and verteporfin significantly suppressed DTP proliferation and induced apoptosis in vitro. In xenograft models, the combination therapy delayed tumor regrowth after treatment cessation compared to imatinib monotherapy.

**Conclusions:**

YAP activity was elevated in GIST DTPs, and YAP inhibitors effectively suppressed this activity. The combination of imatinib and YAP inhibitors enhanced tumor growth suppression. These findings underscore the pivotal role of YAP in DTP survival and demonstrate the therapeutic potential of combining imatinib with YAP inhibitors.

## Introduction

Gastrointestinal stromal tumors (GISTs) are the most common mesenchymal tumors of the gastrointestinal tract [1]. A majority of GISTs harbor gain-of-function mutations in *KIT*, rendering KIT signaling inhibition a highly effective therapeutic strategy [2]. Imatinib, a tyrosine kinase inhibitor (TKI) targeting KIT and PDGFRA, has revolutionized the treatment of GISTs [3]. Among various molecularly targeted therapies for cancer, imatinib has shown remarkable efficacy in advanced GIST, achieving a progression-free survival of approximately 30 months [4]. However, a major challenge with imatinib therapy is the high recurrence rate observed upon treatment discontinuation, even after prolonged periods of clinical benefit. This suggests the development of drug resistance, which remains a significant hurdle [5, 6]. This phenomenon implies that GISTs may enter a drug-tolerant state during imatinib treatment. Supporting this, our previous studies have identified a residual population of viable cells in GIST specimens resected following imatinib therapy [7].

Drug-tolerant persister cells (DTPs) are a small subpopulation of cancer cells that exhibit reversible resistance to therapy through non-genetic mechanisms, potentially acting as precursors to acquired resistance [8–10]. While DTPs have been extensively studied in other malignancies, such as lung cancer, limited data exist on their role in GISTs. To address this gap, we investigated DTPs in GISTs. Our research revealed that imatinib treatment in imatinib-sensitive GIST cell lines induced phosphorylation of FYN and focal adhesion kinase (FAK), which reversed upon treatment cessation [11]. A subset of cells survived in a dormant state during imatinib exposure but resumed proliferation and regained sensitivity to imatinib once the drug was withdrawn [7]. This reversibility suggests that GISTs evade therapy through non-genetic, reversible mechanisms rather than through genetic mutations conferring resistance.

Yes-associated protein (YAP), along with its co-activator transcriptional coactivator with a PDZ-binding domain (TAZ), is a transcriptional regulator that shuttles between the cytoplasm and nucleus. YAP/TAZ activates transcription by interacting with TEAD in the nucleus, while phosphorylation sequesters YAP/TAZ in the cytoplasm or leads to their degradation [12, 13]. Recent studies have highlighted the critical role of YAP in the survival of DTPs, particularly in EGFR-mutant non-small cell lung cancer (NSCLC), where YAP activation suppresses the expression of pro-apoptotic BMF, enabling apoptosis evasion [14]. In addition, activation of the FAK-YAP/TEAD signaling axis is essential for DTP survival in NSCLC, potentially interacting with the tumor microenvironment [15]. In GIST, YAP expression has been reported as an independent prognostic factor in cases that underwent curative resection. The involvement of the FBXW7-YAP pathway has been suggested as a potential molecular mechanism [16]. However, since studies about DTPs in GIST are limited, the role of YAP in GIST DTPs remains unclear.

This study aimed to elucidate the role of YAP in GIST DTPs and assess the therapeutic potential of YAP inhibitors for targeting these cells. By advancing our understanding of the molecular mechanisms underlying DTP survival, we aim to develop strategies that enable the discontinuation of TKI therapy, contributing to curative treatments for GISTs.

## Materials and methods

### Cell lines

The established human GIST cell line, GIST-T1 (Cosmobio, Tokyo, Japan), was used in this study. This *KIT* exon 11-mutant cell line, characterized by a heterozygous 57-base deletion, is sensitive to imatinib [17]. GIST-T1 cells were cultured in Dulbecco’s modified Eagle’s medium supplemented with 10% fetal bovine serum (GE Healthcare, Little Chalfont, Buckinghamshire, UK), 100 U/mL penicillin, and 100 μg/mL streptomycin (Invitrogen, Carlsbad, CA). Cultures were maintained at 37 °C in a humid atmosphere with 5% CO_2_. Cells were treated with imatinib mesylate (Tocris, Bristol, UK), verteporfin (a YAP inhibitor (Selleck chemicals, Houston, TX), or XAV-939 (a tankyrase inhibitor that indirectly suppresses YAP activity; Selleck chemicals, Houston, TX) [18].

### Generation of DTPs and treatments

For DTPs generation, 5 × 10^5^ cells were seeded in 10 cm dish culture dishes and treated with 1 μM imatinib (above the IC_80_ concentration) for ≥ 9 d, with fresh drug added every 3 d. Unless otherwise specified, verteporfin and XAV-939 were added to DTP cultures for 3 d.

### Western blotting analysis

Western blotting was conducted as described previously [19]. Antibodies used included c-KIT (D13A2, #3074), phospho-c-KIT (Tyr703, #3073), YAP (D8H1X, #14074), and phospho-YAP (Ser127, #13008). The secondary antibody, horse-radish peroxidase-conjugated goat anti-rabbit IgG, was also obtained from Cell Signaling Technology (Danvers, MA).

For nuclear and cytoplasmic protein extraction, a Nuclear and Cytoplasmic Extraction Kit (Thermo Fisher Scientific) was used. Harvested cells were suspended in cytoplasmic extraction reagents, centrifuged, and the supernatant (cytoplasmic extraction) was carefully discarded. The remaining pellet was resuspended in nuclear extraction reagent, centrifuged, and the supernatant (nuclear fraction) was collected for analysis.

### Fluorescent staining

Cells were cultured on multi-well glass-bottom dishes (D141400; MATSUNAMI, Osaka, Japan) and fixed with 4% paraformaldehyde for 20 min at 20 to 25 °C. Fixed cells were permeabilized and blocked for 30 min in phosphate-buffered saline containing 0.1% saponin and 3% bovine serum albumin. Cells were incubated with a primary antibody for YAP/TAZ (D24E4, #8418; Cell Signaling Technology, Danvers, MA) for 2 h, followed by incubation with a secondary antibody (Goat anti-Rabbit IgG (H + L) Cross-Adsorbed Secondary Antibody, Alexa Fluor™ 488; Thermo Fisher Scientific, Rockford, IL) for 1 h. Images were obtained using a confocal laser scanning microscope (FLUOVIEW FV3000, Olympus, Tokyo, Japan).

### Cell proliferation assay

For the chemosensitivity assay, cells were seeded in 96-well plates at a density of 2.0 × 10^3^ cells per well and incubated for 24 h. Cells were then exposed to varying concentrations of imatinib, verteporfin, or XAV-939. Proliferation was assessed using the WST-8 assay (2-(2-methoxy-4-nitro-phenyl)-3-(4-nitrophenyl)-5-(2,4-disulfophenyl)-2H-tetrazolium, monosodium salt) (Cell Counting Kit-8, Dojindo Laboratories, Kumamoto, Japan), and absorbance was measured at 450 nm using a microplate reader (iMark; Bio-Rad Laboratories, Hercules, CA, USA).

For the long-term proliferation assay, cells were seeded in 96-well plates at a density of 1.0 × 10^3^ cells per well and incubated for 24 h. Cells were treated with imatinib for 9 d, followed by one of the following conditions for an additional 3 d: (1) imatinib alone, (2) imatinib + 1 µM verteporfin, (3) imatinib + 2 µM XAV-939. These concentrations were determined based on the results of the proliferation assay of verteporfin and XAV-939 in DTPs. After treatment, drugs were washed out, and confluency was evaluated using the Cell Counting Kit-8.

## Caspase 3/7 activity measurements

Caspase 3/7-Glo Assay Kits (Promega Corporation, Madison, WI) were used to measure caspase activity with 2.0 × 10^3^ cells per replicate according to the manufacturer's instructions. The caspase 3/7-Glo assay was performed using a microplate luminometer (Centro LB960, Berthold Technologies, Bad Wildbad, Germany).

### Cell-cycle assay

Cells were fixed in cold 70% ethanol at -20 °C for 2 h. After centrifugation at 300 × g for 5 min, the supernatant was discarded. The pellet was resuspended in 5 μL of Cell Cycle Solution Blue (Dojindo laboratories), followed by incubation at 37 °C for 15 min. Cell cycle analysis and sorting were performed using a FACS Canto II instrument (BD Biosciences, San Jose, CA). Data were analyzed using FlowJo v10 software (BD Biosciences).

### Apoptosis analysis

Cells were harvested and resuspended in 85 µL of binding buffer, followed by the addition of 10 µL of Annexin V-FITC (Medical & biological laboratories, Tokyo, Japan). DAPI was used to stain necrotic cells, as verteporfin interferes with the wavelength of PI. After a 15-min incubation at room temperature, 5 µL of DAPI was added, and the stained cells were analyzed via flow cytometry.

### GIST cell xenograft mouse models

To evaluate the efficacy and safety of verteporfin in vivo, GIST-T1 xenograft mouse models were used. GIST T1 cells were implanted in BALB/cAJcl-nu/nu mice obtained from CLEA Japan, Inc (5 weeks old, male, Tokyo, Japan). For cell inoculation, 1 × 10^7^ cells were injected subcutaneously into the flanks of the mice. When the tumor volume reached approximately 500 mm^3^, the mice were randomly divided into each group based on tumor size. Treatment was administered to four groups: (1) Vehicle (n = 4), (2) verteporfin (10 mg/kg) for 3 d (n = 4), (3) imatinib (100 mg/kg) for 12 d (n = 4), and (4) imatinib (100 mg/kg) for 9 d followed by a combination of imatinib (100 mg) and verteporfin (10 mg/kg) for 3 d (n = 4). Imatinib was administered intraperitoneally every day for 12 days. Verteporfin was administered intraperitoneally on days 10 and 12, with its dosage determined based on a previous report [20]. Body weight and tumor volume were measured every day until day 35. Tumor volumes were determined by measuring the length (L) and width (W) and were calculated as (W^2^ × L)/2.

### Patients

In this retrospective study, 12 patients with GIST who underwent curative resection following preoperative imatinib treatment and 19 patients who underwent curative resection without preoperative treatment between 2004 and 2020 were included. In addition, we expanded the cohort by including recurrent GIST cases: 5 patients who responded to imatinib and 7 patients with imatinib-resistant disease. Data on patient characteristics and histological examination results were retrieved from medical records.

### Immunohistochemistry

The expression of c-KIT and YAP was examined using immunohistochemistry (IHC) staining on formalin-fixed, paraffin-embedded human cancer tissues. Anti-c-KIT (D13A2, #3074) and anti-YAP (D8H1X, #14,074) antibodies from Cell Signaling Technology (Danvers, MA) were used, following previously described protocols [21]. For analysis of subcutaneously implanted tumors, the tissues were embedded in paraffin and subjected to IHC staining for c-KIT and YAP. The ratio of nuclear YAP-positive cells was quantified using the Analysis application (BZ-H3C, KEYENCE, Osaka, Japan). First, nuclei were extracted using the analysis application. Next, up to 20 areas judged to be positive for YAP staining were manually selected to train the software for color recognition. The ratio of nuclear YAP-positive cells was then calculated based on these parameters. Terminal dUTP nick-end labeling (TUNEL) assays (TUNEL Assay Apoptosis Detection Kit, Biotium, Fremont, CA) were performed according to the manufacturer's instructions.

### Statistical analysis

Data are presented as mean ± standard deviation (SD) for both in vitro and in vivo experiments unless otherwise stated. A two-sided Student’s t-test was used to determine statistically significant differences between two groups, while the Tukey–Kramer honestly significant difference test was applied for comparisons among three or more groups. Statistical significance was set at p < 0.05. All statistical analyses were performed using JMP software (version 17.0; SAS Institute, Cary, NC, USA).

## Results

### Reversible nuclear localization of YAP in GIST DTPs

**Fig. 1:**
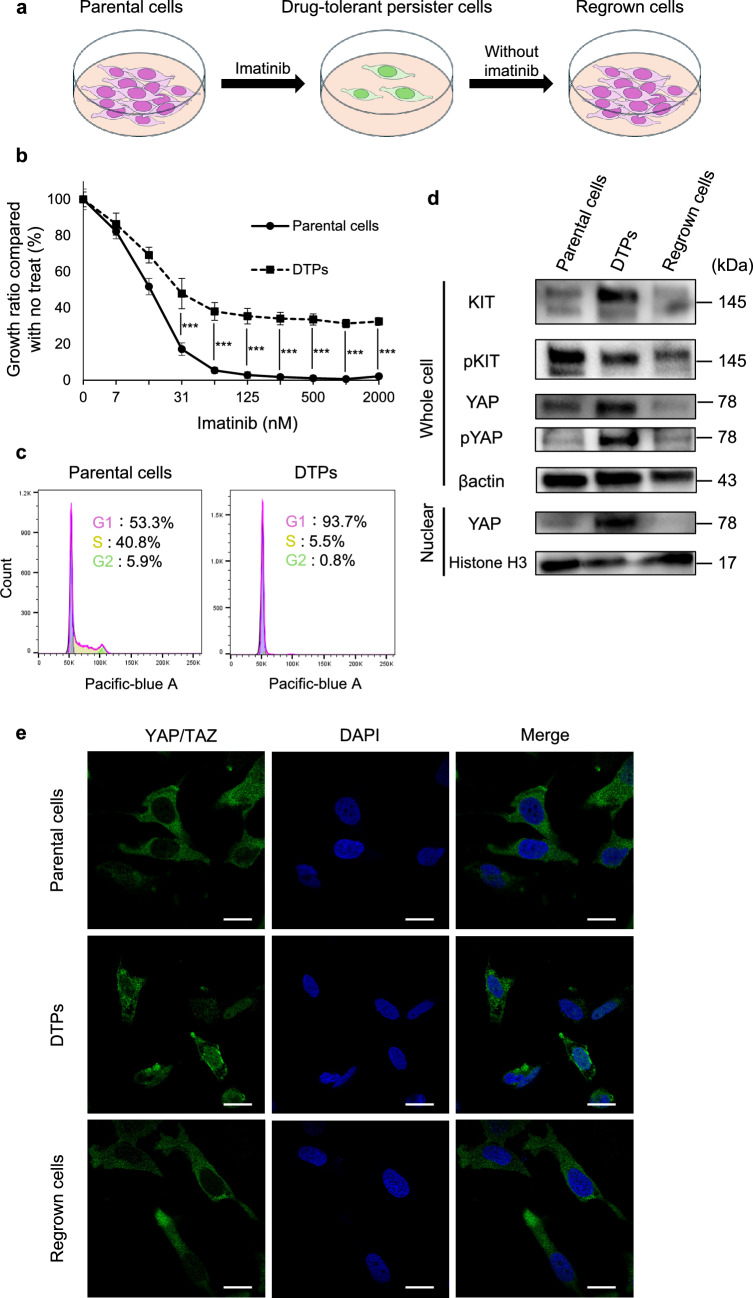
Characterization of imatinib-derived gastrointestinal stromal tumor (GIST) drug-tolerant persister cells (DTPs). **A** Schematic explanation of DTP generation. **B** Cell proliferation measured by WST-8 assays at 72 h after imatinib treatment in GIST-T1 parental cells and DTPs. Each value is presented as mean ± SD (n = 6). Two-sided t-test (***p < 0.001). **C** Western blot analysis of cKIT, pKIT, YAP and pYAP in nuclear and whole cell lysates of parental cells, DTPs, and regrown cells. **D** Cell cycle analysis of parental cells and DTPs. **E** Immunofluorescence staining of YAP/TAZ in parental cells, DTPs, and regrown cells. Scale bar:20 μm

The imatinib-sensitive GIST-T1 cell line was treated with 80% inhibitory concentrations of imatinib to generate DTPs (Fig. 1a). Compared to parental cells, DTPs exhibited reduced sensitivity to imatinib (Fig. 1b) and an increased proportion of cells in the G1 phase, as shown via cell cycle analysis (Fig. 1c). These observations are consistent with our previous findings and existing literature, confirming the successful generation of DTPs. Western blotting revealed decreased levels of phosphorylated KIT and increased total KIT levels in DTPs, indicating inhibition of the KIT pathway by imatinib. Nuclear-cytoplasmic fractionation followed by western blotting showed increased nuclear YAP expression in DTPs (Fig. 1d), and fluorescence immunostaining corroborated enhanced nuclear localization of YAP in these cells (Fig. 1e). Notably, culturing DTPs for 1 week after imatinib washout resulted in decreased nuclear YAP localization, suggesting that the increased YAP activity observed in DTPs was reversible (Fig. 1d, e). This reversibility indicates that the observed changes in YAP activity were not due to genetic mutations but likely involved reversible, non-genetic mechanisms.

### Increased proportion of YAP-positive tumor cell nuclei in samples of patients with GIST following preoperative imatinib treatment

**Fig. 2:**
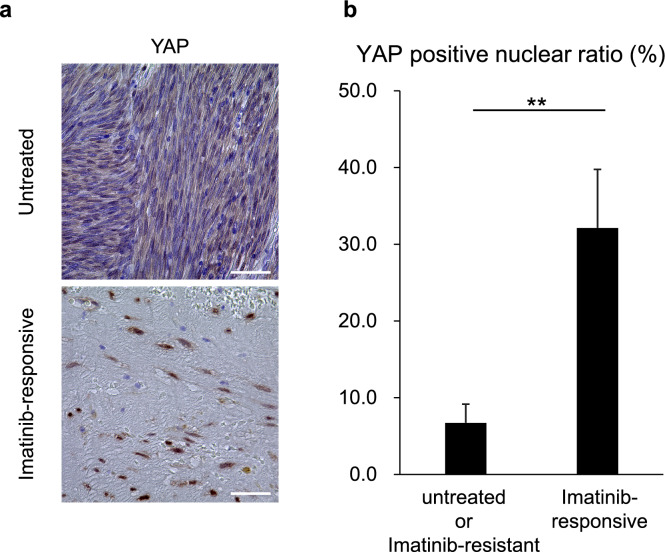
Evaluation of YAP in patient samples **A** immunohistochemistry (IHC) staining for YAP in GIST patient samples with or without preoperative imatinib treatment. Scale bar:50 μm. **B** Proportion of YAP-positive tumor cell nuclei in 26 untreated or imatinib-resistant cases and 17 imatinib-responsive cases. Each value is presented as mean ± SEM. Mann–Whitney U test (**p < 0.01)

Residual tumors that responded to chemotherapy were considered clinical DTPs. Using IHC, we compared YAP activity between resectable GIST cases treated with preoperative imatinib and untreated GIST cases. In addition, we evaluated cases in which imatinib was administered for unresectable or recurrent GIST, followed by surgical resection of either responsive or resistant lesions. Patients’ characteristics are shown in Table 1. In the imatinib-responsive cases, a small number of viable tumor cells were observed (Fig. 2a). The proportion of YAP-positive tumor cell nuclei was significantly higher in imatinib-responsive cases than that in untreated or imatinib-resistant cases (Fig. 2b). In recurrent GIST, imatinib-responsive cases tended to show a higher proportion of YAP-positive tumor cell nuclei than resistant cases, although the difference was not statistically significant (Supplementary Fig. 1). These findings align with the cell-based experiments, where increased YAP activity was also observed in DTPs.

**Table 1:** Patients’ characteristics with or without preoperative imatinib

	Imatinib (-), *n* = 19	Imatinib (+), *n* = 12
Age, years, median (IQR)	70 (62–81)	68 (57–70)
Sex		
Male	7	6
Female	12	6
Tumor location		
Stomach	17	5
Duodenum	2	2
Rectum	0	5
Duration of neo-adjuvant imatinib, months, median (IQR)	–	5.9 (4.0–9.2)
Best resoponse		
Partial response	–	6
Stable disease	–	6
Maximum tumor diameter, mm, median (IQR)	36 (26–61)	73 (62–105)
Mitotic index, per 50 HPFs, median (IQR)	3 (2–9)	0 (0–3)
mFletcher classification		
Very low	1	0
Low	10	0
Intermidiate	2	0
High	6	12
Mutational status		
*KIT exon 11*	10	1
*PDGFRA exon 18*	5	0
Wild type *KIT, PDGFRA*	4	0
Unknown	0	11

### YAP inhibitors reduced nuclear localization of YAP in DTPs

**Fig. 3:**
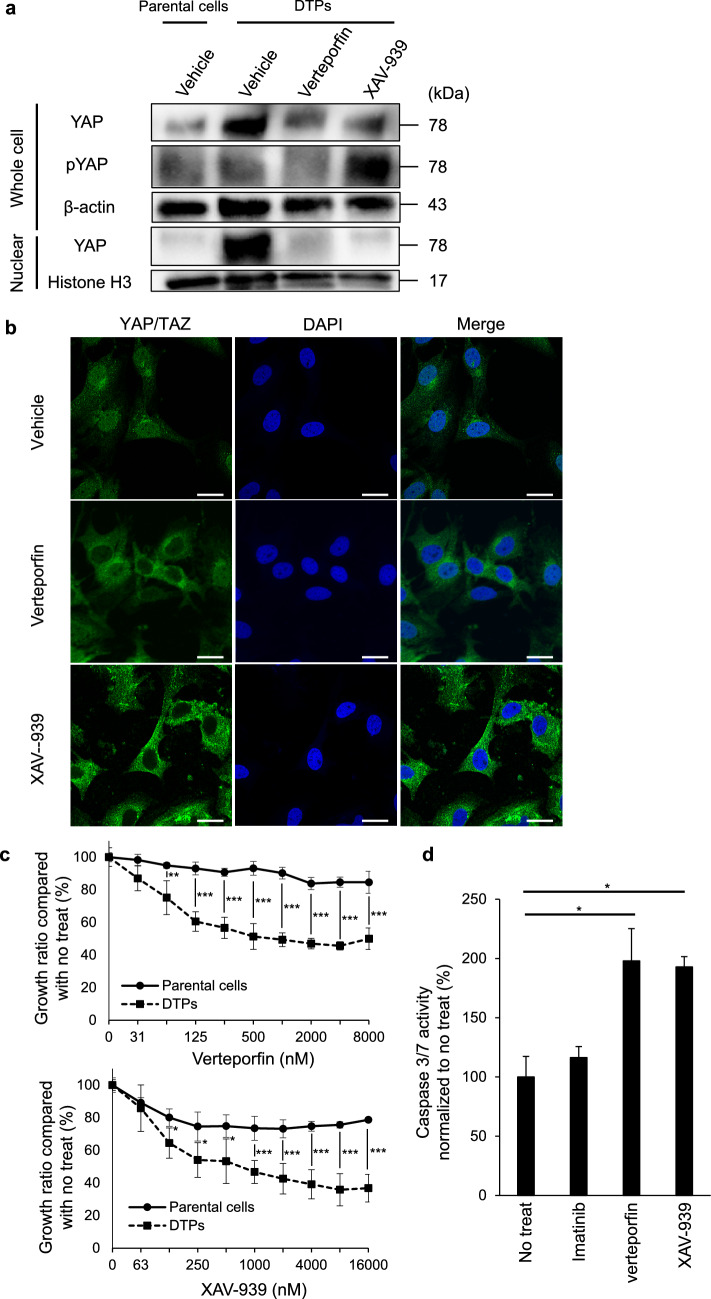
Effects of YAP inhibitors on DTPs. **A** Western blot analysis of YAP and pYAP in nuclear and whole-cell lysates of parental cells, DTPs, and DTPs treated with verteporfin or XAV-939. **B** Immunofluorescence staining of YAP/TAZ in DTPs treated with verteporfin or XAV-939. Scale bar:20 μm. **C** Cell proliferation measured by WST-8 assays at 72 h after verteporfin or XAV-939 treatment in parental cells and DTPs. Each value is presented as mean ± SD (n = 6). Two-sided t-test (*p < 0.05, **p < 0.01, ***p < 0.001). (**D**) Caspase 3/7 assay of the DTPs at 8 h after treatment with DMSO, imatinib, verteporfin or XAV-939, respectively. Each value is presented as mean ± SD (n = 4). Two-sided t-test (*p < 0.05)

Administration of YAP inhibitors to DTPs resulted in a marked decrease in nuclear YAP expression (Fig. 3a). In addition, pYAP expression was remarkably increased in DTPs treated with XAV-939 compared with vehicle-treated cells, whereas verteporfin treatment reduced nuclear YAP without a marked increase in pYAP. Fluorescence immunostaining confirmed reduced nuclear localization of YAP in DTPs following YAP inhibitor treatment (Fig. 3b). These findings demonstrate that YAP inhibitors effectively suppressed the elevated YAP activity characteristic of DTPs. Additionally, sensitivity tests revealed that DTPs exhibited higher sensitivity to YAP inhibitors than parental cells (Fig. 3c), and YAP inhibitors significantly increased the caspase activity in DTPs (Fig. 3d).

### Effect of combination therapies with imatinib and YAP inhibitors on DTPs

**Fig. 4:**
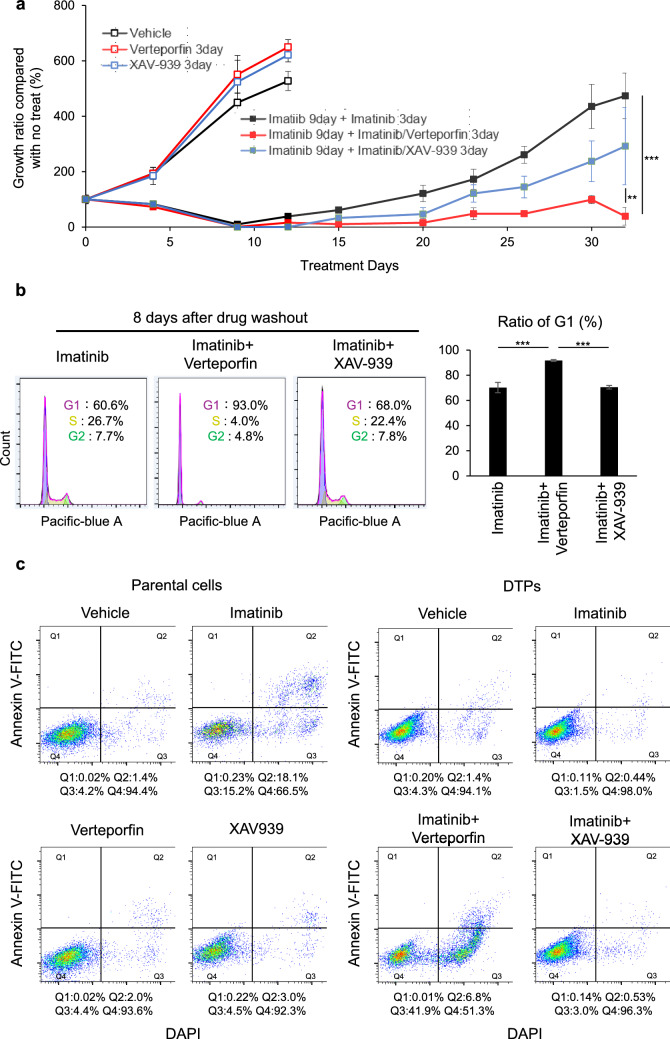
Effects of combination therapies of imatinib and YAP inhibitors. **A** Proliferation of cells treated with the following therapies, followed by drug washout: (1) vehicle, (2) verteporfin for 3 d, (3) XAV-939 for 3 d, (4) imatinib for 12 d, (5) imatinib for 9 d followed by a combination of imatinib and verteporfin for 3 d, and (6) imatinib for 9 d followed by a combination of imatinib and XAV-939 for 3 d. Each value is presented as mean ± SD (n = 6). Tukey–Kramer honestly significant difference test (**p < 0.01, ***p < 0.001). **B** Cell cycle assay of DTPs treated with the indicated therapies and incubated for 8 d after drug washout: (1) imatinib for 3 d, (2) imatinib and verteporfin for 3 d, and (3) imatinib and XAV-939 for 3 d. Each value is presented as mean ± SD (n = 3). Two-sided t-test (***p < 0.001). **C** Annexin V-FITC/DAPI apoptosis assay in parental cells and DTPs treated with the indicated therapies for 8 h. Parental cells: (1) vehicle, (2) imatinib, (3) verteporfin, (4) XAV-939. DTPs: (5) vehicle, (6) imatinib, (7) imatinib and verteporfin, and (8) imatinib and XAV-939

The efficacy of combination therapies with imatinib and YAP inhibitors were evaluated. GIST cells were treated for 12 d, followed by drug washout and a cultivation period of 3 weeks to evaluate the long-term effects. Imatinib monotherapy led to immediate cell proliferation upon drug removal, whereas combination therapies demonstrated sustained inhibition of cell proliferation during the observation period. Among the YAP inhibitors, verteporfin exhibited stronger inhibitory effects on cell proliferation than XAV-939 (Fig. 4a). Notably, treatment with YAP inhibitors alone did not suppress cell proliferation, indicating their specific efficacy in targeting DTPs. Cell cycle analysis showed that most cells remained in the G1 phase 8 d after drug washout when treated with the combination of imatinib and verteporfin (Fig. 4b). Imatinib induced apoptosis in parental cells but not in DTPs. However, combination therapy with imatinib and verteporfin successfully induced apoptosis in DTPs (Fig. 4c). These findings suggest that the combination therapy of imatinib and verteporfin is an effective approach for targeting DTPs.

### Antitumor effects of the combination therapy with imatinib and verteporfin in GIST xenograft models

**Fig. 5:**
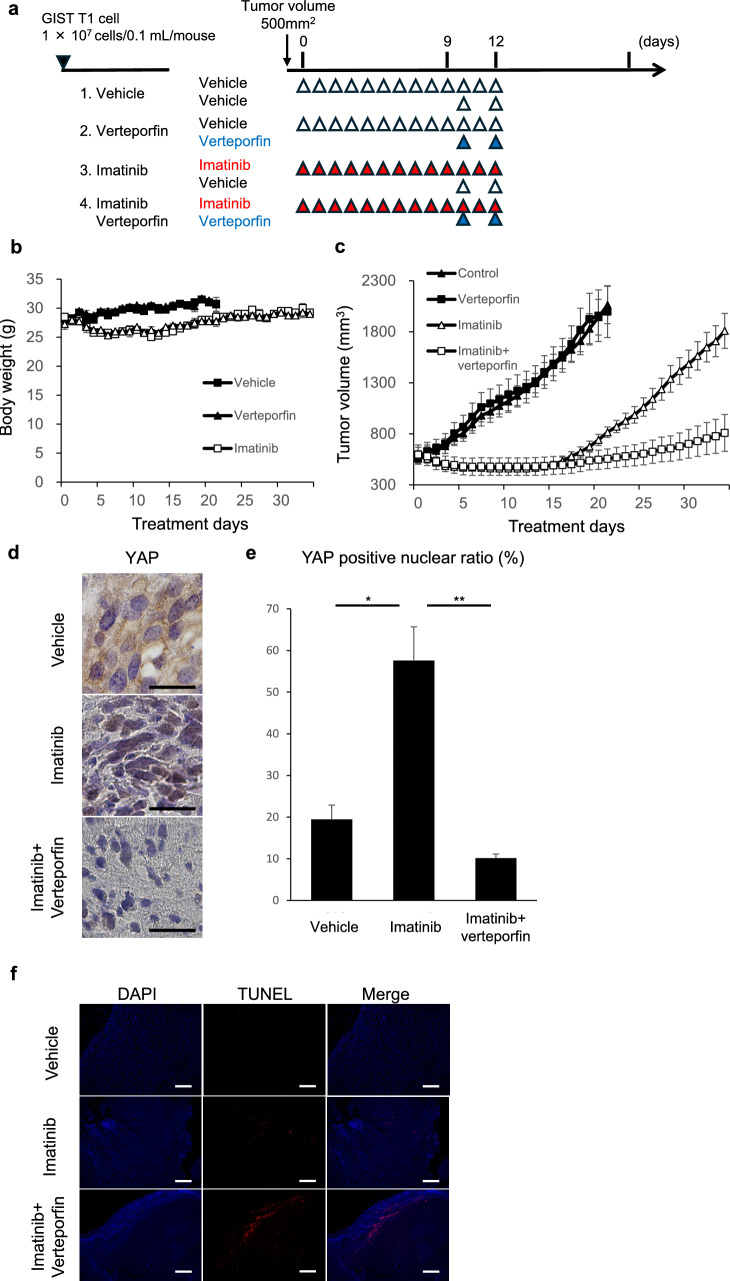
Antitumor effects of verteporfin in a GIST xenograft mouse model. **A** GIST-T1 cells were transplanted subcutaneously and treated with the following therapies: (1) vehicle (n = 4), (2) verteporfin for 3 d (n = 4), (3) imatinib for 12 d (n = 4), and (4) imatinib for 9 d followed by a combination of imatinib and verteporfin for 3 d (n = 4). Treatment was initiated when tumor volume reached approximately 500 mm^3^ (day 0). **B** Body weight and (**C**) tumor volume were measured every day until day 35. Each value is presented as mean ± SD (n = 4). Two-sided t-test (*p < 0.05, **p < 0.01, ***p < 0.001). **D** Representative YAP-stained tumor sections from each treatment group. Scale bar: 25 μm. **E** Proportion of YAP positive nuclei. Each value is presented as mean ± SEM (n = 3), Tukey–Kramer honestly significant difference test (*p < 0.05, **p < 0.01). **F** Apoptosis analysis by TUNEL staining from each treatment group. Scale bar: 100 µm

To further investigate the tumor growth inhibitory effects of combination therapy with imatinib and verteporfin, a GIST-T1 xenograft mouse model was used. Mice were treated with verteporfin alone, imatinib alone, or a combination of imatinib and verteporfin (Fig. 5a). Weight loss was observed in the imatinib-treated groups, but body weight recovered after treatment cessation. Verteporfin treatment did not result in weight loss (Fig. 5b). Compared to imatinib monotherapy, the combination therapy significantly suppressed tumor regrowth after treatment cessation (Fig. 5c). Verteporfin monotherapy did not exhibit any inhibitory effects on tumor growth. To evaluate the effect of verteporfin on YAP activity, additional IHC analyses immediately after the treatments were performed on mice treated under the same conditions (Fig. 5a). Imatinib monotherapy increased the proportion of YAP-positive tumor cell nuclei, while the combination therapy of imatinib and verteporfin reduced the proportion (Fig. 5d and 5e). In TUNEL assay, imatinib monotherapy induced cellular apoptosis, and the combination therapy of imatinib and verteporfin more strongly induced it. Cellular apoptosis was particularly induced in areas adjacent to the necrotic regions (Fig. 5f).

## Discussion

Drug-tolerant persister cells (DTPs) are a subpopulation of cancer cells that survive therapeutic pressure through reversible, non-genetic mechanisms. In contrast to acquired resistance driven by genetic alterations, such as secondary *KIT* mutations, DTPs evade apoptosis and enter a transient drug-tolerant state without harboring permanent genomic changes [8–10]. In our previous work, we demonstrated that secondary *KIT* mutations were observed only in GIST-T1 cells subjected to long-term imatinib exposure, but not in cells exposed for a short duration [22], supporting the idea that early-phase resistance is mediated by non-genetic adaptations. Consistent with this, the present study revealed that YAP activity was markedly upregulated in DTPs following imatinib treatment, as evidenced by increased nuclear localization. Notably, this change was reversible upon drug withdrawal, further indicating the plastic, non-genetic nature of the DTP state. These findings suggest that GIST cells can evade imatinib-induced apoptosis by dynamically reprogramming their gene expression profiles, with YAP functioning as a key regulator of this adaptive survival response. Although previous reports have described the association between YAP overexpression and malignancy in GIST [16], our study observed the reversible changes in YAP expression in GIST DTPs. This aspect highlights the novelty of our findings.

The findings of this study suggest that YAP activation plays a key role in the survival of DTPs during molecular-targeted therapy in GIST. In imatinib-sensitive GIST cells, DTPs induced by imatinib treatment exhibited increased nuclear localization and activity of YAP. YAP inhibitors demonstrated significant efficacy in targeting these cells. Moreover, the combination of imatinib and verteporfin sustained tumor growth suppression for up to 3 weeks following drug withdrawal, highlighting the potential utility of this combined approach in overcoming DTPs. Clinical data further supported these findings, showing an increased proportion of YAP-positive nuclei in preoperatively treated samples that responded well to imatinib, consistent with observations from cell-based experiments. These results address a persistent therapeutic challenge in GIST, where complete tumor eradication remains elusive despite advances in molecular-targeted therapies.

The relationship between DTPs and YAP has been reported in several preclinical and clinical studies of NSCLC [14, 15], suggesting that YAP activation following molecular-targeted therapy may represent a conserved phenomenon across diverse cancer types. In NSCLC-derived DTPs with EGFR or ALK mutations, targeted inhibitors have been shown to activate FAK signaling, which promotes nuclear localization of YAP [15]. In our previous study, we observed elevated FAK expression in GIST DTPs induced by imatinib [11]. These findings collectively provide indirect evidence that FAK may also be involved in the nuclear translocation of YAP in GIST DTPs. However, the present study did not directly investigate the mechanistic link between FAK and YAP activation, leaving it uncertain whether FAK signaling plays a causal role in YAP activation in this context. Considering the established role of FAK in NSCLC, it is logical to propose that FAK may similarly contribute to YAP-mediated resistance in GIST. Future studies are needed to validate this hypothesis and elucidate the underlying mechanisms.

Inhibition of KIT by imatinib has been shown to upregulate the pro-apoptotic factor BIM through transcriptional and post-translational mechanisms in GIST cell lines [23]. Conversely, DTPs induced by EGFR/MEK inhibition in EGFR-mutant NSCLC evade apoptosis by suppressing the expression of the pro-apoptotic factor BMF via the YAP/TEAD/SLUG complex [14]. Both BIM and BMF are involved in mitochondrial apoptosis, suggesting that YAP activation in DTPs may interfere with imatinib-induced cell death by modulating intrinsic apoptotic pathways [24]. Consistent with these findings, this study demonstrated that DTPs under imatinib treatment avoided apoptosis, whereas verteporfin effectively induced apoptosis in these cells. Notably, YAP inhibitors were ineffective in parental GIST cells, likely because cells primarily relied on the KIT signaling pathway. In contrast, DTPs exhibited a dependency on YAP for apoptosis evasion, underscoring the therapeutic potential of YAP inhibitors in this context.

In this study, two YAP inhibitors—verteporfin and XAV-939—were used, and both agents suppressed YAP activity in DTPs. However, the differences in pYAP expression observed in Fig. 3a likely stem from their distinct mechanisms of action. Verteporfin directly inhibits YAP/TAZ and YAP/TEAD interactions [25, 26], while XAV-939 inhibits tankyrase, stabilizing AMOT family proteins [18]. AMOT family proteins have been shown to activate LATS kinase, leading to YAP phosphorylation [27]. This mechanistic difference may account for the observed increase in pYAP expression in DTPs treated with XAV-939. Simultaneous inhibition of KIT and YAP in GIST represents a promising strategy to induce apoptosis in DTPs, potentially leading to complete tumor eradication.

The combination of imatinib and YAP inhibitors demonstrated significant efficacy in vitro and vivo. While imatinib monotherapy resulted in tumor regrowth after treatment cessation, verteporfin addition significantly delayed regrowth. IHC revealed an increased proportion of YAP-positive nuclei in tumors immediately after imatinib monotherapy, whereas combination therapy reduced this proportion. These findings confirm the establishment of DTPs in the animal model and highlight verteporfin’s inhibitory effects on YAP. However, the reduction in tumor volume observed in vivo was modest compared to the dramatic decrease in cell numbers seen in vitro, suggesting that imatinib’s in vivo efficacy is limited. This limitation may explain the eventual tumor regrowth observed in the combination therapy group. In this study, imatinib was administered for 12 d and verteporfin for only 3 d to align with in vitro protocols. Clinically, preoperative imatinib is typically administered for over 3 months, during which viable cells are rarely observed in well-responding cases. Extending the duration of drug administration in future animal studies could potentially yield different outcomes. Additionally, optimization of verteporfin dosing regimens is warranted.

In the verteporfin-only group, no significant weight loss was observed, while weight loss in the combination group was attributed primarily to imatinib, with no additional effects from verteporfin. This indicates that verteporfin can be safely administered in mice. Verteporfin, though primarily known as a YAP inhibitor, is clinically approved as a photosensitizer for photodynamic therapy in age-related macular degeneration [28]. While patients had to avoid direct sunlight for 48 h post-treatment, with eye and skin protection recommended, no serious adverse events were reported in randomized trials for age-related macular degeneration treatment [29, 30]. Its established safety profile and clinical use render verteporfin an attractive candidate for early adoption in cancer therapy, provided its antitumor effects are further validated.

This study has some limitations. First, our experiments were conducted using only one GIST cell line due to the unavailability of other imatinib-sensitive lines in Japan. Future studies utilizing additional GIST cell lines, including patient-derived samples, are needed to confirm these findings. Second, the limitation of this study is the lack of functional validation through YAP knockdown or knockout experiments. Although such approaches would provide strong support for the results obtained with YAP inhibitors, they were technically challenging to perform in DTPs. This is because DTPs represent a rare and transient cell population that emerges only under continuous exposure to molecular-targeted therapy, such as imatinib, making efficient gene delivery and selection difficult. The limited cell numbers, slow proliferation, and the need to maintain drug pressure during the process further complicate genetic manipulation. Future studies employing alternative strategies—such as establishing inducible gene silencing systems prior to DTP induction—may help overcome these technical challenges and enable direct functional assessment of YAP depletion in DTPs. Third, defining DTPs in clinical specimens poses challenges. Residual cells in cases responding to preoperative imatinib were classified as DTPs, but the possibility of including imatinib-resistant cells cannot be excluded, potentially confounding the results. Examining *KIT* mutation status pre- and post-treatment may aid in more accurate identification of DTPs. Lastly, while this study demonstrated the effectiveness of combination therapy with imatinib and verteporfin, the optimal YAP inhibitor for GIST treatment remains to be determined. Although YAP-dependent growth has been observed in malignant mesothelioma and solid tumors with neurofibromatosis 2 mutations, the American Association for Cancer Research recently reported on the efficacy of the oral YAP/TEAD inhibitor VT3989 in a phase 1 trial. Exploring other YAP inhibitors like VT3989 could contribute to optimizing combination therapies in GIST treatment.

This study demonstrated that YAP activity is elevated in DTPs following imatinib treatment in imatinib-sensitive GIST. Our findings suggest that combining imatinib with the YAP inhibitors may effectively target DTPs and reduce recurrence rates. By addressing the survival mechanisms of DTPs, this study provides a foundation for developing combination therapies to overcome resistance and facilitate the discontinuation of TKI therapy. These findings contribute to advancing curative treatment strategies for GIST and highlight the need for continued investigation into the molecular pathways underpinning drug tolerance.

## Data Availability

The datasets generated/analyzed during the current study are not publicly available because they contain private information pertaining to the research participants but are available on request from the corresponding author.
